# Prevalence of microvascular complications among patients with type 2 diabetes mellitus who visited diabetes clinics in Saudi Arabia

**DOI:** 10.15537/smj.2023.44.2.20220719

**Published:** 2023-02

**Authors:** Mounther M. Alnaim, Abdullah Alrasheed, Abdullah A. Alkhateeb, Mohammed M. Aljaafari, Abdulrahman Alismail

**Affiliations:** *From the Family Medicine Department (Alnaim), Primary Health Care, King Abdulaziz Hospital, Ministry of National Guard Health Affair; from the College of Medicine (Alkhateeb, Aljaafari, Alismail), King Faisal University, Al Ahsa; from the Family Medicine Department (Alrasheed), Primary Health Care, King Abdulaziz Hospital, Ministry of National Guard Health Affair, Dammam, Kingdom of Saudi Arabia.*

**Keywords:** microvascular complications, type 2 diabetes mellitus, prevalence

## Abstract

**Objectives:**

To determine the microvascular complications prevalence in patients with type 2 diabetes mellitus (DM) in the eastern province of the Kingdom of Saudi Arabia.

**Methods:**

The participants in this retrospective, cross-sectional study included patients with type 2 DM who visited the diabetes clinics of primary health care centers of 2 National Guard Hospitals, Eastern Province, Saudi Arabia.

**Results:**

This study included 935 patients with type 2 diabetes (54.1% women versus 45.9% men). Oral hypoglycemic medication was the most common treatment (90.3%). Overall, 55.1% of patients visited the ophthalmology clinic for retinopathy screening. The last glycated hemoglobin A1c result (mean: 8.04%) was higher than the second-to-last result (mean: 8.03%), or the third-to-last result (mean: 7.99%). The prevalence of microvascular complications of DM was 55.1%. Independent significant factors linked with a higher risk of microvascular issues of DM were higher age, visits to an ophthalmology clinic, and the use of injection therapy. The most typical complications that our patients experienced was nephropathy (80.2%), followed by retinopathy (32.7%), and neuropathy (8.4%).

**Conclusion:**

Microvascular complications were extremely common in type 2 DM patients in our region. Being older, regularly visiting an ophthalmologist, and using injection therapy were predictive factors correlated with a higher chance of experiencing these complications.

**D**iabetes mellitus (DM) is a chronic condition that occurs when the pancreas of the human body cannot produce enough insulin, or the body cannot use it. This leads to an inadequate amount of blood glucose in the body.^1^ According to the International Diabetes Federation, 463 million people have diabetes globally, half of whom are undiagnosed, which can lead to severe complications.^2^ According to the World Health Organization’s data on the rate of DM, Saudi Arabia is the second highest country in the Middle East and the seventh highest in the world with approximately 7 million people with diabetes.^3^

Risk factors for developing type 2 DM include a family history of diabetes, ethnicity, older age, previous gestational diabetes, unhealthy diet, excess body fat, obesity, physical inactivity, and smoking.^1^ Complications associated with DM are either macrovascular or microvascular. Macrovascular complications of DM include ischemic heart, cerebrovascular, and peripheral vascular diseases. Microvascular complications of DM include retinopathy, neuropathy, and nephropathy. These complications can lead to myocardial infarction, stroke, foot ulcers, amputations, blindness, sexual dysfunction, and renal failure.^4.6^

Only a few studies have focused on the prevalence of microvascular complications of type 2 DM. A cross-sectional study carried out in India showed that of 200 participants, 26 had diabetic nephropathy. Among these participants, 12% had microalbuminuria and 1% had macroalbuminuria.^7^ Another study carried out in Arar, Saudi Arabia including 276 participants showed that 5.8% of patients had diabetic nephropathy, 6.5% had renal insufficiency, and 1.4% had chronic kidney failure.^8^ A cross-sectional study carried out in Ahmednagar, India between October 2017 and March 2018 reported that the prevalence of diabetic neuropathy in diabetic patients was 6.3% for pure neuropathy and 42.7% for patients with significant clinical examination findings only.^9^ A cross-sectional study of 187 recruited patients with type 2 DM in Al-Khobar, Saudi Arabia showed the prevalence of diabetic peripheral neuropathy was 37.4%.^10^ Another study conducted in Al Ahsa in 2009 revealed that 30% of 473 diabetic participants had diabetic retinopathy.^11^

Although most patients with type 2 DM develop microvascular complications, we found a lack of data regarding the prevalence of microvascular complications in type 2 DM in the eastern province of Saudi Arabia. Therefore, as there is high prevalence of type 2 DM in our community, we aimed to determine the rate of microvascular complications among diabetics in our community.

## Methods

This retrospective, cross-sectional study was carried out in the Primary Health Care Diabetes Mellitus Clinics at King Abdulaziz Hospital and Imam Abdulrahman bin Faisal Hospital, National Guard Health Affairs, Eastern Province, Saudi Arabia. King Abdullah International Medical Research Center approved this study, and the study follows the principles of the Helsinki Declaration. All available patients known to have type 2 DM who visited the clinics between March 2021 and May 2021 were selected on a target date, and the sample obtained was larger than our target sample size. Patients with type 1 DM and those with macrovascular complications were excluded.

The data were collated directly from the original database using a questionnaire with chart review from the diabetes clinics of primary health care centers of two National Guard Hospitals in the Eastern Province, Saudi Arabia. The data included the patients’ primary demographic data, onset of DM, last 3 glycated hemoglobin A1c (HbA1c) results, patient complaints of microvascular complications, onset of microvascular complications, management plan, and visits to the ophthalmology clinic for a retinopathy screening test in the past 3 years. We used the last 3 HbA1c results to assess patients’ compliance with their treatment plan and determine whether the treatment affected the prevalence of DM complications. We used 3 years as the cut-off point because the electronic system of the National Guard Health Affairs (Bestcare system) has been implemented for only approximately 3-4 years.

Microvascular complications were defined as retinopathy diagnosed by an ophthalmologist and documented in the patient file during the annual retinopathy screening. Nephropathy was defined as a persistent decrease in the glomerular filtration rate <60 or persistent urinary albumin ≥30 mg/day or equivalent for ≥3 months. Neuropathy was defined as the loss of pinprick, vibration, and temperature sensation by examination or documentation of painful neuropathic symptoms in the patient file.

### Statistical analysis

The total sample size was 400, as specified by the Richard Geiger equation, and the margin of error was 5% with a confidence of 95%. Magnitudes of central tendency and dispersion were used to analyze continuous (numerical) variables. All categorical (nominal) data are presented as numbers and percentages. The relationship between microvascular complications of DM and patient baseline characteristics was assessed using the Chi-square test (categorical data) or independent sample t-test (continuous data). Based on the significant results, a subsequent multivariate regression model was used to determine the independent significant factors associated with microvascular complications of DM with a corresponding odds ratio (OR) and 95% confidence interval (CI). Two-tailed analysis with a *p*-value of <0.05 was used as a cut-off value for statistical significance. All data analyses were performed using the Statistical Package for Social Sciences for Windows, version 26 (IBM Corp., Armonk, NY, USA).

## Results

**Figure 1:**
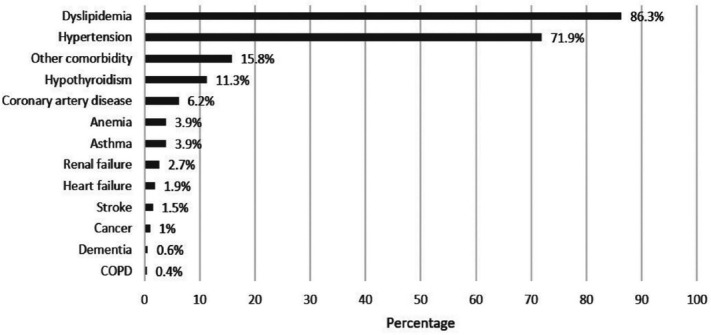
- Patients’ associated comorbidity. COPD: chronic obstructive pulmonary disease

**Figure 2:**
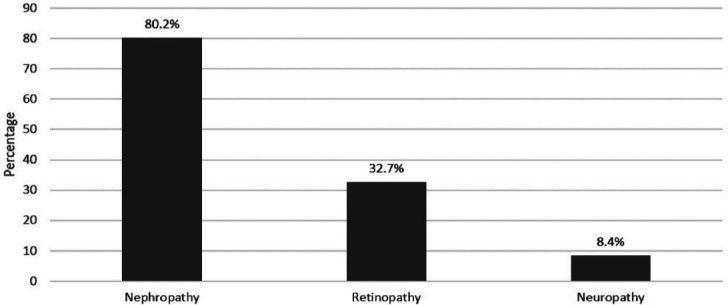
- Microvascular complications of type 2 diabetes mellitus.

We analyzed the data of 935 patients with type 2 DM. As shown in Table 1, the most common age group was 56-65 years (33.9%), with more than half (54.1%) being females and most visiting the National Guard Hospital, Al Ahsa (77.4%). The majority of patients were Saudis (99.7%). The percentage of obese patients was 69.8%, with nearly all patients being diagnosed with DM for ≤3 years (93.4%). The rate of patients who complained of microvascular complications was 52.3%. Of these, 73% had been diagnosed within the past 3 years. The percentage of patients who visited the ophthalmology clinic for retinopathy checkups was 55.1%. Oral hypoglycemic medication was the most common treatment (administered to 90.3% of patients). In addition, the respective mean values of the last, second-to-last, and third-to-last HbA1c results were 8.04%, 8.03%, and 7.99%, respectively. The most commonly associated comorbidity was dyslipidemia (86.3%), followed by hypertension (71.9%), and hypothyroidism (11.3%) (Figure 1). As shown in Figure 2, the most commonly diagnosed microvascular complications were nephropathy (80.2%) and retinopathy (32.7%).

**Table 1:** - Baseline characteristics of the patients with type 2 diabetes mellitus (N=935).

Variable	n (%)
***Age group***	
≤45 years	83 (08.9)
46–55 years	269 (28.8)
56–65 years	317 (33.9)
>65 years	266 (28.4)
***Gender***	
Male	429 (45.9)
Female	506 (54.1)
***National Guard ospital location***	
Al Ahsa	724 (77.4)
Dammam	211 (22.6)
***Nationality***	
Saudi	932 (99.7)
Non-Saudi	03 (0.30)
***Body mass index***	
Normal (18.5–24.9 kg/m^2^)	57 (06.1)
Overweight (25–29.9 kg/m^2^)	225 (24.1)
Obese (≥30 kg/m^2^)	653 (69.8)
***Onset of DM***	
<3 years	62 (06.6)
>3 years	873 (93.4)
***Patient complaints of microvascular complications***	
Yes	489 (52.3)
No	446 (47.7)
***Onset of microvascular complications of DM (n=489)***	
<3 years	132 (27.0)
>3 years	357 (73.0)
***Patient visited an ophthalmology clinic for retinopathy screening***	
Yes	515 (55.1)
No	420 (44.9)
Management plan*	
Lifestyle modification	505 (54.0)
Oral hypoglycemic medication	844 (90.3)
Injection therapy	401 (42.9)
	Mean±SD
Last HbA1c result	8.04±2.83
Second-to-last HbA1c result	8.03±2.72
Third-to-last HbA1c result	7.99±3.14

* Variable with multiple responses. HbA1c: glycated hemoglobin A1c, SD: standard deviation, DM: diabetes mellitus

When evaluating the relationship between microvascular complications of DM and the baseline characteristics of the diabetic patients, we found a significant relationship between microvascular complications of DM according to age group (*p*<0.001), the National Guard Hospital location (*p*<0.001), patients who underwent retinopathy screening (*p*<0.001), lifestyle modifications (*p*<0.001), injection therapy (*p*<0.001), and second-to-last HbA1c result (*p*=0.030) (Table 2).

**Table 2:** - Relationship between microvascular complications of DM and the baseline characteristics of the patients with type 2 DM (N=935).

Factor	Yes (n=489)	No (n=446)	*P*-value^§^
***Age group***			
≤55 years	148 (30.3)	204 (45.7)	<0.001**
>55 years	341 (69.7)	242 (54.3)	
***Gender***			
Male	219 (44.8)	210 (47.1)	0.481
Female	270 (55.2)	236 (52.9)	
***National Guard hospital location***			
Al Ahsa	331 (67.7)	393 (88.1)	<0.001**
Dammam	158 (32.3)	53 (11.9)	
***BMI***			
Normal (18.5–24.9 kg/m^2^)	27 (05.5)	30 (06.7)	0.246
Overweight (25–29.9 kg/m^2^)	128 (26.2)	97 (21.7)	
Obese (≥30 kg/m^2^)	334 (68.3)	319 (71.5)	
***Onset of DM***			
<3 years	27 (05.5)	35 (07.8)	0.153
>3 years	462 (94.5)	411 (92.2)	
***Patient visited an ophthalmology clinic for retinopathy screening***			
Yes	326 (66.7)	189 (42.4)	<0.001**
No	163 (33.3)	257 (57.6)	
Management plan*			
Lifestyle modification	227 (46.4)	278 (62.3)	<0.00**
Oral hypoglycemic medication	435 (89.0)	409 (91.7)	0.157
Injection therapy	252 (51.5)	149 (33.4)	<0.00**
	Mean±SD	Mean±SD	*p*-value^‡^
Last HbA1c result	8.09±1.71	7.97±3.69	0.506
Second-to-last HbA1c result	8.22±3.41	7.83±1.63	0.030**
Third-to-last HbA1c result	7.99±1.63	8.01±4.23	0.900

Values are presented as number and percentage (%).

* Variable with multiple responses.

§ The p-value was calculated using the Chi-square test.

‡ The *p*-value was calculated using the independent sample t-test.

** Significant at *p*<0.05.

HbA1c: glycated hemoglobin A1c, SD: standard deviation, DM: diabetes mellitus, BMI: body mass index

In the multivariate regression model (Table 3), older age (>55 years), retinopathy screening, and the use of injection therapy as a treatment method were independent significant predictors of an increased risk of microvascular complications of DM, whereas visiting the National Guard Hospital in Al Ahsa was an independent factor for a decreased risk of microvascular complications of DM. This finding further indicates that the risk of microvascular complications in the older age group (>55 years) was predicted to increase by at least 1.8-fold compared to the younger age group (≤55 years) (adjusted OR [AOR]=1.764, 95% CI: 1.320–2.356, *p*<0.001). Patients who visited an ophthalmology clinic for retinopathy screening had a 2.4-fold increased risk of microvascular complications of DM compared to those who did not visit the clinic (AOR=2.408, 95% CI: 1.800–3.222, *p*<0.001). Similarly, patients who used injection therapy as a treatment method had a 2.2-fold increased risk of microvascular complications of DM compared to those who did not use injection therapy (AOR=2.219, 95% CI: 1.638–3.006, *p*<0.001). However, patients who were admitted to the National Guard Hospital, Al Ahsa had a 60% lower risk of developing microvascular complications of DM compared to those who were admitted to the National Guard Hospital in Dammam (AOR=0.410, 95% CI: 0.267–0.630, *p*<0.001). Other variables included in the model did not show any significant effect on microvascular complications of DM after regression adjustment, including lifestyle modifications and the second-to-last HbA1c result (*p*>0.05).

**Table 3:** - Multivariate regression analysis of the independent significant factors associated with microvascular complications of DM (N=935)

Factor	AOR	95% CI	*P*-value
***Age group***			
≤55 years	Ref		
>55 years	1.764	1.320–2.356	<0.001**
***National Guard hospital location***			
Al Ahsa	0.410	0.267–0.630	<0.001**
Dammam	Ref		
***Patient visited an ophthalmology clinic for retinopathy screening***			
No	Ref		
Yes	2.408	1.800–3.222	<0.001**
***Lifestyle modification***			
No	Ref		
Yes	0.786	0.566–1.093	0.152
***Injection therapy***			
No	Ref		
Yes	2.219	1.638–3.006	<0.001**
Second-to-last HbA1c result	0.949	0.875–1.029	0.207

** Significant at *p*<0.05, AOR: adjusted odds ratio, CI: confidence interval, HbA1c: glycated hemoglobin A1c, ref: reference

## Discussion

This study investigated the prevalence of microvascular complications of DM and determined their associated risk factors in a Saudi diabetic cohort. Our results showed a high prevalence of microvascular complications in patients with DM. More than half (52.3%) of our sample population complained of the occurrence of these complications. A similar prevalence of microvascular complications has been reported in a study from India^12^ which included 390 patients with type 2 DM at the Rural Health and Training Centre of Sri Ramachandra Medical College. According to previous reports, the prevalence of microvascular diseases is 52.1% higher than that of macrovascular complications (29.7%). In China,^13^ a study reported that the 5-year prevalence of microvascular complications was 57.5% higher than the prevalence of macrovascular complications (51.4%), but lower than the prevalence of vascular complications in general (73.2%). In Sudan,^14^ the prevalence of microvascular complications among patients with type 2 DM was 45.9%, which was also in accordance with our results. However, in India,^7^ the incidence of microvascular complications, specifically nephropathy, was only 13% lower. Necessary measures to control the progression of diabetes are vital to prevent its complications.

Older age was associated with an increased risk of microvascular complications. This finding is consistent with that reported by Li et al^13^ who reported that the odds of microvascular complications increased with age but decreased with education level. In our study, the odds of complications decreased in patients admitted to the National Guard Hospital, Al Ahsa. However, the true effect of disease progression on patient admission between the 2 centers (National Guard Hospital in Al Ahsa versus National Guard Hospital in Dammam) cannot be determined by a single study. Therefore, further investigation is required to establish the true effect.

There was modest compliance among our patients in terms of visits to ophthalmologists for retinopathy checkups. The odds of microvascular complications increased in this group of patients, likely due to the fact that these patients realized the effects of disease progression, which led them to consult an ophthalmologist. However, the duration of DM did not appear to affect disease progression related to the microvasculature. A study conducted among diabetic patients in Pakistan^11^ reported that diabetic retinopathy showed a significant association with diabetes duration, uncontrolled blood glucose level, hyperlipidemia, and hypercholesterolemia, whereas in India,^9,12^ an increased odds of microvascular complications were related to hypertension and uncontrolled HbA1c levels. In our study, however, we discovered that an increased HbA1c level was associated with an increased risk of microvascular complications of DM, specifically the second-to-last HbA1c result. Therefore, glycemic control should be considered at all times to prevent and minimize the occurrence of complications.

Diabetic nephropathy was the most dominant complication of diabetes identified in our patients (80.2%), followed by retinopathy (32.7%) and neuropathy (8.4%). These findings are consistent with those reported by Ali et al^15^ who found that among patients with poor glycemic control (HbA1c >6.5%), 68.5% had neuropathy, 56.2% had nephropathy, and 31.4% had retinopathy. In addition, even in newly diagnosed diabetic patients who had uncontrolled glycemic control, the frequency of microvascular complications was higher than that in those who had average glycemic control (HbA1c <6.5%). The prevalence of neuropathy due to DM (44.9%) was also higher in a study conducted in India,^12^ followed by nephropathy (12.1%) and diabetic foot (7.2%). However, in a systematic review by Bekele et al,^1^ retinopathy, nephropathy, metabolic syndrome, impotence, and depression were the most common microvascular complications found in diabetic Ethiopians. The authors echoed the growing number of cases of microvascular disease in the diabetic population in their country. Effective monitoring of these individuals is necessary to reduce the incidence of diabetes progression and complications.

Adherence to the therapeutic plan for DM is also an important factor in reducing the complications of diabetes. Hence, the dominant use of oral hypoglycemic medication was evident in our results, wherein 90.3% of our patients used this type of therapy, 54% opted for lifestyle modification, and 42.9% used injection therapy. Effective intervention and management are concomitant with disease prevention as reported by Kulkarni and Ganvir^9^ who concluded that controlling blood glucose levels, modifying ones’ lifestyle, engaging in physical activity, and managing ones’ diet could play a major role in reducing the risk of DM complications.

### Study limitation

The main limitation of this study was the inability to obtain patient data that was more than 3 years old because not all patient data were transferred to the new electronic system. In addition, this study only involved 2 centers in the eastern region of Saudi Arabia. We look forward to overcoming these limitations in future studies. Despite these limitations, this study will help researchers to correlate the results of this study with their findings and help doctors understand the prevalence of microvascular complications in the eastern region of Saudi Arabia.

In conclusion, there was a high prevalence of microvascular complications in patients with type 2 DM in our region. Being older, regularly visiting an ophthalmologist, and using injection therapy were predictive factors that increased the risk of microvascular complications. Furthermore, nephropathy due to DM was the major microvascular complication identified among the patients, followed by retinopathy and neuropathy. The prevalence of microvascular complications among patients with diabetes can be decreased by constant monitoring and follow-up. Thus, healthcare providers play a vital role in ensuring that patients with DM comply with clinic visits and adhere to their therapeutic plans.
